# Increasing medication adherence and income assistance access for first-episode psychosis patients

**DOI:** 10.1371/journal.pone.0179089

**Published:** 2017-06-07

**Authors:** Jason Randall, Dan Chateau, James M. Bolton, Mark Smith, Laurence Katz, Elaine Burland, Carole Taylor, Nathan C. Nickel, Jennifer Enns, Alan Katz, Marni Brownell, the PATHS Equity Team

**Affiliations:** 1 Department of Community Health Sciences, Max Rady College of Medicine, Rady Faculty of Health Sciences, University of Manitoba, Winnipeg, Manitoba, Canada; 2 Manitoba Centre for Health Policy, Department of Community Health Sciences, Max Rady College of Medicine, Rady Faculty of Health Sciences, University of Manitoba, Winnipeg, Manitoba, Canada; 3 Department of Psychiatry, Max Rady College of Medicine, Rady Faculty of Health Sciences, University of Manitoba, Winnipeg, Manitoba, Canada; 4 Department of Family Medicine, Max Rady College of Medicine, Rady Faculty of Health Sciences, University of Manitoba, Winnipeg, Manitoba, Canada; Maastricht University, NETHERLANDS

## Abstract

**Background:**

Assertive community treatment for first-episode psychosis programs have been shown to improve symptoms and reduce service use. There is little or no evidence on whether these programs can increase access to income assistance and improve medication adherence in first episode psychosis patients. This research examines the impact of the Early Psychosis Prevention and Intervention Service (EPPIS) on these outcomes.

**Methods:**

We extracted data on EPPIS patients held in the Data Repository at the Manitoba Centre for Health Policy. The Repository is a comprehensive collection of person-level de-identified administrative records, including data from Manitoba’s health services. We compared income assistance use and antipsychotic medication adherence in EPPIS patients to a historical cohort matched on pattern of diagnosis. Confounders were adjusted through propensity-score weighting with asymmetrical trimming. Odds ratios (OR), hazard ratios (HR) and 95% confidence intervals were calculated.

**Results:**

We identified a matched sample of 244 patients and 449 controls. EPPIS patients had a higher rate of income assistance use during the program (67·4% vs. 38·7%; p< 0·0001). EPPIS patients were more likely to have been prescribed at least one antipsychotic medication than the control cohort, both during the program (OR = 15·05; 95%CI 10·81 to 20·94) and after the program ended (OR = 5·20; 95%CI: 4·50 to 6·02). Patients in EPPIS were also more likely to adhere to their medication during the program (OR = 4·71; 95%CI 3·75 to 5·92), and after the program (OR = 2·54; 95%CI 2·04 to 3·16).

**Conclusion:**

Enrolment in the EPPIS program was associated with increased adherence to antipsychotic medication treatment and improved uptake of income assistance.

## Introduction

Early intervention for psychosis and Assertive Community Treatment (ACT) programs have become increasing popular methods in the treatment of psychotic disorders [1–7]. These programs tend to focus on individuals with a first episode of psychosis and use a comprehensive treatment approach that combines medication, psychotherapy, and other resources. Generally, the approach in assertive community treatment is to engage patients in active treatment outside of the hospital [8,9]. Family members are often involved in the treatment process in order to increase their awareness and ability to cope with and respond to the symptoms of the patient. Previous research has provided evidence that these programs are effective at improving symptoms and prognosis [4,7,10–14].

Lack of medication adherence among those with psychotic disorders is associated with poorer outcomes [15–17]. Improving medication adherence is one way that first-episode interventions can improve prognosis. Individuals with schizophrenia are disproportionally of low socio-economic status [18]. The cause of this has not yet be conclusively determined, but schizophrenia may cause decreased SES due to the work and social impairments caused by the disorder [19]. Low SES can prevent people from affording their medication. However, income assistance and other welfare programs that subsidize drug costs may increase adherence to medication. Therefore, research assessing how well these programs impact on adherence and access to government programs that help provide medications is needed.

### Early Psychosis Prevention and Intervention Service (EPPIS)

The Early Psychosis Prevention and Intervention Service (EPPIS) was established in Winnipeg, Manitoba, Canada in 2003 as an ACT treatment program for first-episode psychosis patients. This program targets individuals aged 13 to 35 with a recent onset of psychotic symptoms within a single-payer integrated healthcare system with a population of about 650 000 people. Similar to other first-episode programs, EPPIS emphasizes adherence to medication and psychotherapy (including group therapy with family members and other clients), with the goal of promoting and maintaining social connectedness. EPPIS staff also work with guidance counsellors, employers, and other key individuals in the client’s environment, to assist with re-integration of clients into their communities. This may involve community psychoeducation to reduce the stigma of psychotic disorders, and/or developing flexible work and educational environments.

Referrals are made directly to the EPPIS program by clinicians, at which time a Mental Health Clinician is assigned. If further follow-up is required, clients are assigned to a psychiatrist within one working day. An initial interview is conducted within 7 days of referral; however, cases considered urgent are seen within 3 working days. Cases of immediate risk are referred to the emergency department or a mobile crisis team. The initial interview is conducted in a non-clinical setting if necessary. At the initial interview, information is collected about basic demographics, personal history and experience, and assessment of general psychiatric symptoms. The Mental Health Clinician assigned at referral remains the clinician throughout the remainder of treatment in the program. Antipsychotic medication is considered a primary form of therapy in this program. However, clients retain the ability to refuse medication and still remain in the program. The program not only treats the individual client but aims to provide support for their family as well.

This study used a unique population-based dataset of social and medical records to examine whether EPPIS affected medication use and access to income assistance. The specific outcomes assessed were medication prescriptions received, medication adherence, and receipt of income assistance. We hypothesized that the assertive nature of this program would improve medication outcomes and increase the use of available social assistance programs.

## Methods

### Data source

This study used data from the PATHS Data Resource in the Population Health Research Data Repository housed at the Manitoba Centre for Health Policy (MCHP)[20]. The Repository contains the de-identified administrative health records for Manitoba’s publicly-funded health system representing virtually the entire provincial population with the exception of individuals who are incarcerated or federal military officials. The Repository data are linkable at the individual level across multiple service areas, including hospital discharge abstracts, physician billing submissions, pharmaceutical prescriptions from the Drug Programs Information Network (DPIN), and services provided by the Manitoba Adolescent Treatment Centre (MATC)–which contains data for the Winnipeg EPPIS program. Data for inpatient service use is coded in ICD—9-CM up to March 31, 2004, and in ICD-10-CA after this date. Physicians’ services are billed using ICD-9-CM. These data contain information on physician treatment both inside and outside of hospital Pharmaceutical prescriptions are coded using Anatomical Therapeutic Chemical (ATC) classification codes or Generic Drug names. SES is derived using the average income of each census dissemination area (DA). Each DA contains between 400 and 700 people, providing neighborhood-level income. Data on income assistance was collected through the Social Assistance Management Information Network. People in Manitoba are eligible for income assistance if they need help meeting basic personal and family needs; this includes those who have a mental illness that is likely to last more than 90 days which prevents them from meeting their basic needs.

### Study design

A matched-cohort design was used in this study. Individuals enrolled in the EPPIS program were identified using data provided by MATC which was responsible for administrating the program for the period assessed by this study. Individuals with 3+ visits in the program were considered to have been treated by the program. Data were available for the calendar years 2003 to 2012. Controls were then identified through matching for diagnostic pattern of psychotic disorders. EPPIS patients’ records were assessed for two years prior to program entrance for the occurrence of psychosis diagnoses. Matches were required to have received all of the same psychosis diagnosis codes but not have been treated by the program. In order to reduce differences between controls and cases, propensity scores were calculated using logistic regression and conditional probabilities for treatment were derived [21]. These conditional probabilities were used to determine inverse probability of treatment weights [21]. Asymmetric trimming was performed at the 2·5^th^ and 97·5^th^ percentiles to reduce the effect of individuals with extreme weights [22]. Trimming is necessary to remove outliers from the EPPIS and control groups that could bias the results. Confounders were selected using the High Dimensional Propensity Score algorithm that detected variables that were associated with both treatment and outcome [23]. Standardized differences were assessed to determine whether confounder variables were successfully adjusted through weighting [24]. Treated individuals were compared to the matched cohort during the period of treatment (up to 24 months) and after treatment. The control sample was drawn from a time period before the EPPIS program (1998–2001). We were unable to find a control group of sufficient size that was similar to the client group within the EPPIS program period, possibly due to the program successfully enrolling its entire target population. Regions of the province not covered by EPPIS are not directly comparable due to large differences in health service availability and the lack of another major city in the province.

### Descriptive statistics

Descriptive statistics for age, sex, SES (grouped into quintiles), and follow-up length (years) were derived for both cohorts. For those in the program, information on total time in treatment (days) and number of visits to the treatment program were derived T-tests were used to determine if treatment days and visits differed significantly between low (quintiles 1 and 2) and high (quintiles 3–5) income groups.

### Outcomes

The first outcome was the use of income assistance. A binary variable was derived from these data for each group for the two-year period before treatment, during treatment and for a two-year period following the end of treatment, which identified individuals who were receiving income assistance at some point during those periods. A binary variable for ever being on income assistance after treatment was also derived. Time on income assistance was derived by measuring the years on income assistance per person year.

The other outcomes pertained to the use of and adherence to antipsychotic medications (including depot medications) and included any first-or-second generation antipsychotic medications (ATCs -'N05AH', 'N05AX', 'N05AD', 'N05AB', 'N05AF', 'N05AA') at any dosage. The DPIN database was the data source for these outcomes We determined (i) whether individuals had ‘received any prescription for an antipsychotic medication’, and ii) whether individuals had ‘antipsychotic medication adherence’; which was defined as having a medication possession ratio (i.e., days of medication received divided by days in treatment period) of at least 80% in a year [25]. These outcomes both refer specifically to outpatient dispensation of antipsychotics. Medications administered within inpatient units are not included in this study.

### Statistical analysis

Logistic regression and Cox proportional hazards regression analyses were used to estimate odds ratios (OR), hazard ratios (HR) and 95% confidence intervals (95%CI). Analyses were performed for the treatment period, and during post-treatment. The post-treatment analysis was performed using two methods. First, ORs were determined for a two-year period following discharge from the program. Then HRs were estimated using all available follow-up years for each individual, without restriction to the two-year period. Age was included as a covariate in all of the regression models. Baseline use of income assistance was also included as a covariate for the income assistance analysis. Inverse probability of treatment weighting was applied so that the results would estimate the average treatment effect (ATE). Statistical analysis was performed using SAS 9.3 (SAS Institute, Cary NC).

### Ethics

Approval was obtained from the University of Manitoba’s Health Research Ethics Board (HREB #: H2011:294) and the Government of Manitoba’s Health Information Privacy Commission (HIPC #- 2013/2014–22). The study used de-identified administrative health data to protect the health information of those in the sample.

## Results

MATC records contained data on 284 EPPIS patients, and an initial matched control group of 1036 individuals was identified based on diagnostic pattern. Trimming resulted in a sample of 244 cases and 449 controls. See Table 1 for the ICD codes and patient characteristics that were identified as potential confounders through their correlation with exposure status and the outcome, and controlled through propensity score weighting. These conditions are expected to be related to first-episode psychosis and poor mental health. Skin disorders and use of ectoparasiticides, for example, might be coded due to the development of delusions around parasite infestations. Refraction/accommodation disorders might be coded when individuals report vague visual disturbances. After weighting, the standardized differences for these variables were all below 0·1 with the exception of macrolides and lincosamides which was 0·10. This indicates good adjustment for these covariates [26].

**Table 1:** Confounders adjusted for in propensity score model.

	Standardized Differences^a^	
Confounder	Before	After
Age at Index Date	0.348280192	0.009049535
Biological Sex	0.219947494	0.02661594
Income Quintile 1—Lowest	0.108475699	0.008593202
Income Quintile 2	0.013325061	0.028948717
Income Quintile 3	0.045699607	0.015316186
Income Quintile 4	0.004724669	0.01442587
Income Quintile 5—Highest	0.018423922	0.011983356
3019 UNSPECIFIED PERSONALITY DISORDER (Frequent)	0.027302021	0.023982185
3019 UNSPECIFIED PERSONALITY DISORDER (Once)	0.031667492	0.041709179
3059 OTHER, MIXED, OR UNSPECIFIED DRUG ABUSE (Frequent)	0.031824191	0.079360528
3059 OTHER, MIXED, OR UNSPECIFIED DRUG ABUSE (Once)	0.107746162	0.055663443
311 DEPRESSIVE DISORDER, NOT ELSEWHERE CLASSIFIED (Once)	0.089414847	0.038084531
3679 UNSPECIFIED DISORDER REFRACTION,ACCOMMODATION (Frequent)	0.141381309	0.062709814
490 BRONCHITIS, NOT SPECIFIED ACUTE/CHRONIC (Frequent)	0.047025681	0.034252122
7299 OTHER UNSPECIFIED DISORDERS OF SOFT TISSUE (Once)	0.019512613	0.02630248
7099 UNSPECIFIED DISORDER SKIN, SUBCUTANEOUS TISSUE (Once)	0.112108397	0.027953873
3149 UNSPECIFIED HYPERKINETIC SYNDROME (Once)	0.145359288	0.003437781
4722 CHRONIC NASOPHARYNGITIS (Once)	0.045136098	0.001541434
J01F MACROLIDES AND LINCOSAMIDES (Once)	0.029828411	0.101493409
N02A OPIOIDS (Once)	0.185675292	0.014160535
N06A ANTIDEPRESSANTS (Once)	0.120518105	0.046785163
P03A ECTOPARASITICIDES (Frequent)	0.006403249	0.032898485
N04A ANTICHOLINERGIC AGENTS (Once)	0.100542028	0.053046039
N06B PSYCHOSTIMULANTS AND NOOTROPICS (Once)	0.167713541	0.039542159
J07B VIRAL VACCINES (Once)	0.091037297	0.003615597

^a^ standardized difference between groups measured before and after weighting

Frequent notation indicates multiple occurrence of the code. Once indicates a single occurrence of that code.

Descriptive statistics on controls and EPPIS treatment group are shown in Table 2. The sample was 77.5% males with an average age of 18.8. Approximately three-quarters of the sample was between 17 and 24 and only 9 individuals were older than 24. The average follow-up time available was considerably longer for the control group (10·2 years versus 3·1 years due to the use of a historical control with more years of potential administrative data to use). Clients of the program had a mean duration of treatment of 510.4 days (median = 467) with a mean of 98.9 visits. Mean duration of treatment was significantly longer for high income individuals (555.0 days) than low income individuals (452.8; p = 0·019).

**Table 2:** EPPIS and control group demographics and treatment statistics.

		EPPIS		Control	
		N	%	N	%
**Untrimmed sample**		284	21.5	1036	78.5
**Trimmed sample**		244	35.2	449	64.8
**Sex**	Female	55	22.5	145	32.3
	Male	189	77.5	304	67.7
**SES Quintile**	1	60	24.6	132	29.4
	2	46	18.9	87	19.4
	3	45	18.4	75	16.7
	4	36	14.7	67	14.9
	5	43	17.6	76	16.9
	*NF	14	5.7	12	2.7
		**Mean (SD)**	**Median**	**Mean**	**Median**
**Age**		18.8 (2.48)	18.5	19.9 (3.66)	20
**Follow-up (years)**		3.1 (2.6)	2.5	10.2 (3.2)	11
**Treatment period (days)**		510.4 (307.2)	467	NA	
	High income	555.0 (302.5)	547		
	Low income	452.8 (305.1)	361.0		
**Total visits**		98.9 (75.2)	78	NA	
	High income	106.1 (73.9)	83		
	Low income	89.6 (76.2)	69.5		

Table 3 contains descriptive data on the use of income assistance. Before initiation of treatment, the EPPIS group had a slightly higher rate of income assistance (50% versus 41·2%), but this difference increased during the treatment period (67·4% versus 38·7%). Table 4 shows the results of the regression analyses for income assistance use post-treatment. The EPPIS program was associated with a significant increase in the use of income assistance after the treatment period (HR = 2·38, 95%CI 2·06 to 2·74).

**Table 3:** Use of income assistance by EPPIS and control group.

	EPPIS		Control	
**Income assistance**	**N**	**%**	**N**	**%**
before	109	50.0	184	41.2
during	147	67.4	173	38.7
ever	164	75.2	269	60.2
**Time on assistance**	Years*	**SD**	Years*	**SD**
ever post-treatment	0.75	0.3	0.59	0.4

*Years per person year

**Table 4:** Effect of EPPIS on ever using income assistance.

	Ever on Income Assistance		
**Unweighted**			
post treatment	HR (95% CI)	2.46 (2.00 to 3.04)	<.0001
2 year post treatment	OR (95% CI)	3.68 (2.43 to 5.56)	<.0001
**Average Treatment Effect (ATE)**			
post treatment	HR (95% CI)	2.38 (2.06 to 2.74)	<.0001
2 year post treatment	OR (95% CI)	3.49 (2.69 to 4.352)	<.0001

All estimates adjusted for age and pre-treatment IA use in regression model

Table 5 shows the effect of the intervention on outpatient medication use and adherence. During treatment, those in the program were significantly more likely to (i) have used at least 1 antipsychotic prescription (OR = 15·05, 95%CI: 10·81 to 20·94), and (ii) adhered to that medication (OR = 4·71, 95%CI: 3·75 to 5·92). These effects diminished, but were still significant, during the post-treatment period.

**Table 5:** Effect of EPPIS treatment on receiving outpatient medications and medication adherence.

	At least 1 Antipsychotics Prescription			Antipsychotic Adherence		
**Unweighted**						
during treatment	OR (95% CI)	14.25 (8.28 to 24.54)	<.0001	OR (95% CI)	4.59 (3.22 to 6.54)	<.0001
post treatment	HR (95% CI)	5.28 (4.27 to 6.52)	<.0001	OR (95% CI)	2.33 (1.67 to 3.24)	<.0001
**Average Treatment Effect (ATE)**						
during treatment	OR (95% CI)	15.05 (10.81 to 20.94)	<.0001	OR (95% CI)	4.71 (3.75 to 5.92)	<.0001
post treatment	HR (95% CI)	5.20 (4.50 to 6.02)	<.0001	OR (95% CI)	2.54 (2.04 to 3.16)	<.0001

All estimates adjusted for age in regression model

## Discussion

In this study, we evaluated the EPPIS program for its effect on access to income assistance and medication use and adherence. During and after the EPPIS program, clients were more likely than controls to be receiving income assistance. They were also more likely to be prescribed medication and adhere to that medication. Individuals from high-income areas stayed in the treatment program for a longer time period, but did not make more visits to the program than their lower income counterparts.

Previous research on first-episode intervention programs has largely focused on clinical and health service outcomes. Some research has supported these programs as a method of reducing the severity of symptoms during the treatment phase [5]. Individual studies have not consistently found a significant reduction in hospital service use, although a meta-analysis showed reduced hospitalization [6]. There is currently less evidence around the effect of these programs on medication adherence, social service use/income assistance, and their effect on reducing health disparity.

In our study, the use of antipsychotic medication was higher in the treatment group and remained higher after clients left the program. This suggests the program was effective in its goal of initiating and maintaining medication adherence. Poor medication adherence is a risk factor for poor prognosis among individuals with psychotic disorders [15,16]. Adherence to medication has been found to increase the odds of remission and to reduce the time to remission in first episode psychosis patients [15]. Reaching patients that disengage from treatment is an important goal of assertive community treatment models. Being able to demonstrate that patients in the program are using medications at an increased rate is an indication that the program is effective at achieving this goal. Some studies have examined the effect of the program on the use of antipsychotics. Chen et al (2011) found that their first-episode intervention was associated with increased use of second generation antipsychotics [13]. Other studies have found mixed or negative results [27,28]. The finding that EPPIS increased the use of antipsychotics after program discharge is significant and indicates that these programs can alter long-term adherence to medications.

Individuals with psychotic disorders not only have issues maintaining employment, but can also experience difficulty accessing social services available to them. The EPPIS program was associated with an increased use of income assistance. Enrolling in income assistance can not only help the patient in maintaining housing and other personal needs, but also improves their ability to adhere to their pharmaceutical treatment.

The assessment of length of time treated and number of visits suggests that there could still be health disparity among patients of the program. Because we were unable to find a concurrent sample of controls, we cannot determine whether the program actually reduced disparity by income.

### Strengths and limitations

The strengths of this study include the use of a large population-based sample, adjustment for a large variety of confounding factors, objective outcome measures, low levels of attrition, and capture of the entire population treated by EPPIS during the study years. These strengths reduce the likelihood of bias due to selection, attrition, or outcome detection (whether outcome events are more/less likely to be detected based on treatment group). We were also able to link the data across multiple sectors (health, social services) and follow individuals over long periods of time. This improves our ability to control covariates and to detect differences in outcomes.

Limitations include the lack of information about specific symptoms experienced by individual patients, both during the initial assessment of these patients and during treatment. This prevents us from adjusting for the severity of illness through the propensity-score weighting. It is possible that patients with more severe psychotic symptoms were more likely to be enrolled in the EPPIS program. This might have been why finding concurrent controls was difficult. Matching was done based on diagnostic patterns in an attempt to reduce the likelihood of there being a bias due to illness type and severity. However residual confounding may still exist. We were unable to find a group of comparable patients for matching in the time frame that EPPIS was operating. This could bias the results through the influence of secular trends, but expert clinicians in the Winnipeg area believe that EPPIS was the only significant change in treatment during this period. Our inability to locate a concurrent sample of controls may be a limitation to the study, but is also a sign that the program was effective at locating and enrolling its target population. Had there been a large number of eligible patients that were missed during the treatment period, then we should have been able to locate them through similarities in their medical records. Income groups were determined based on census dissemination area and are therefore not assessed individually. This reduces the accuracy of the income quintiles with respect to the individuals involved in this study. There was a parallel income assistance program run by the city of Winnipeg until April of 1999. Information on this program and its clients is not available and may be the cause of the initially low levels of assistance for the control group. However, any potential bias in the regression results due to this would be expected to be towards the null. Adherence and medication use measures in the administrative data cannot confirm that medications were taken properly, only that the medications were dispensed to the patients. The use of expensive antipsychotic medications which are covered by income assistance increases the likelihood that those individuals will be on income assistance. This may introduce some confounding for those outcomes.

In conclusion, this study showed improvements in the treatment measures that we expected the EPPIS program to affect. This study provides additional support for the adoption of assertive treatment services targeted toward first-psychosis patients.
